# Non-invasive ventilation versus high-flow nasal oxygen for postextubation respiratory failure in ICU: a post-hoc analysis of a randomized clinical trial

**DOI:** 10.1186/s13054-021-03621-6

**Published:** 2021-06-28

**Authors:** Arnaud W. Thille, Grégoire Monseau, Rémi Coudroy, Mai-Anh Nay, Arnaud Gacouin, Maxens Decavèle, Romain Sonneville, François Beloncle, Christophe Girault, Laurence Dangers, Alexandre Lautrette, Quentin Levrat, Anahita Rouzé, Emmanuel Vivier, Jean-Baptiste Lascarrou, Jean-Damien Ricard, Keyvan Razazi, Guillaume Barberet, Christine Lebert, Stephan Ehrmann, Alexandre Massri, Jeremy Bourenne, Gael Pradel, Pierre Bailly, Nicolas Terzi, Jean Dellamonica, Guillaume Lacave, René Robert, Stéphanie Ragot, Jean-Pierre Frat, for the HIGH-WEAN Study Group and the REVA research network, Florence Boissier, Delphine Chatellier, Céline Deletage, Carole Guignon, Florent Joly, Morgane Olivry, Anne Veinstein, Dalila Benzekri-Lefevre, Thierry Boulain, Grégoire Muller, Yves Le Tulzo, Jean-Marc Tadié, Adel Maamar, Suela Demiri, Julien Mayaux, Alexandre Demoule, Lila Bouadma, Claire Dupuis, Pierre Asfar, Marc Pierrot, Gaëtan Béduneau, Déborah Boyer, Benjamin Delmas, Bérénice Puech, Konstantinos Bachoumas, Edouard Soum, Séverin Cabasson, Marie-Anne Hoppe, Saad Nseir, Olivier Pouly, Gaël Bourdin, Sylvène Rosselli, Anthony Le Meur, Charlotte Garret, Maelle Martin, Guillaume Berquier, Abirami Thiagarajah, Guillaume Carteaux, Armand Mekontso-Dessap, Antoine Poidevin, Anne-Florence Dureau, Marie-Ange Azais, Gwenhaël Colin, Emmanuelle Mercier, Marlène Morisseau, Caroline Sabatier, Walter Picard, Marc Gainnier, Thi-My-Hue Nguyen, Gwenaël Prat, Carole Schwebel, Matthieu Buscot

**Affiliations:** 1 Centre Hospitalier Universitaire de Poitiers, Service de Médecine Intensive Réanimation, Médecine Intensive Réanimation, 2 rue la Milétrie, 86021 Poitiers Cedex, France; 2 Centre d’Investigation Clinique 1402 ALIVE Research Group, University of Poitiers, Poitiers, France; 3 Centre Hospitalier Régional d’Orléans, Médecine Intensive Réanimation, Orléans, France; 4 Centre Hospitalier Universitaire de Rennes, Service des Maladies Infectieuses et Réanimation Médicale, Hôpital Ponchaillou, Rennes, France; 5 Hôpital Pitié-Salpêtrière, Service de Pneumologie, Médecine Intensive et Réanimation (Département R3S), AP-HP 6 - Sorbonne, INSERM, UMRS1158 Neurophysiologie Respiratoire Expérimentale et Clinique, Sorbonne Université, Paris, France; 6 Hôpital Bichat - Claude Bernard, Médecine Intensive Réanimation, AP-HP, Université Paris Diderot, Paris, France; 7 Centre Hospitalier Universitaire d’Angers, Département de Médecine Intensive Réanimation, Université d’Angers, Angers, France; 8 Centre Hospitalier Universitaire de Rouen, Hôpital Charles Nicolle, Département de Réanimation Médicale, Normandie Université, UNIROUEN, EA3830-GRHV, Institute for Research and Innovation in Biomedicine (IRIB), Rouen, France; 9 Centre Hospitalier Universitaire Félix Guyon, Service de Réanimation Polyvalente, Saint Denis de la Réunion, France; 10 Centre Hospitalier Universitaire de Clermont-Ferrand, Hôpital Gabriel Montpied, Service de Réanimation Médicale, Clermont-Ferrand, France; 11 Centre Hospitalier de la Rochelle, Service de Réanimation, La Rochelle, France; 12 Centre de Réanimation, Centre Hospitalier Universitaire de Lille, Université de Lille, Lille, France; 13 Hôpital Saint-Joseph Saint-Luc, Réanimation Polyvalente, Lyon, France; 14 Médecine Intensive Réanimation, Centre Hospitalier Universitaire de Nantes, Nantes, France; 15 Hôpital Louis Mourier, Réanimation Médico-Chirurgicale, AP-HP, INSERM, UMR IAME 1137, Sorbonne Paris Cité, Université Paris Diderot, Colombes, France; 16 Hôpitaux Universitaires Henri Mondor, Service de Réanimation Médicale DHU A-TVB, AP-HP, Créteil, France; 17 Groupe Hospitalier Régional Mulhouse Sud Alsace, Service de Réanimation Médicale, Site Emile Muller, Mulhouse, France; 18 Service de Médecine Intensive Réanimation, Centre Hospitalier Départemental de Vendée, La Roche Sur Yon, France; 19 Centre Hospitalier Régional Universitaire de Tours, Médecine Intensive Réanimation, CIC 1415, Réseau CRICS-Trigger SEP, Centre d’étude des pathologies respiratoires, INSERM U1100, Université de Tours, Tours, France; 20 Centre Hospitalier de Pau, Service de Réanimation, Pau, France; 21 Centre Hospitalier Universitaire La Timone 2, Médecine Intensive Réanimation, Réanimation des Urgences, Aix-Marseille Université, Marseille, France; 22 Service de Réanimation, Centre Hospitalier Henri Mondor d’Aurillac, Aurillac, France; 23 Centre Hospitalier Universitaire de Brest, Médecine Intensive Réanimation, Brest, France; 24 Centre Hospitalier Universitaire Grenoble Alpes, Médecine Intensive Réanimation, INSERMU1042, HP2, Université Grenoble-Alpes, Grenoble, France; 25 Centre Hospitalier Universitaire de Nice, Médecine Intensive Réanimation, Archet 1, UR2CA, Université Cote d’Azur, Nice, France; 26 Centre Hospitalier de Versailles, Service de Réanimation Médico-Chirurgicale, Le Chesnay, France

**Keywords:** Airway extubation, Ventilator weaning, Acute respiratory failure, Noninvasive ventilation, High-flow nasal oxygen

## Abstract

**Background:**

In intensive care units (ICUs), patients experiencing post-extubation respiratory failure have poor outcomes. The use of noninvasive ventilation (NIV) to treat post-extubation respiratory failure may increase the risk of death. This study aims at comparing mortality between patients treated with NIV alternating with high-flow nasal oxygen or high-flow nasal oxygen alone.

**Methods:**

Post-hoc analysis of a multicenter, randomized, controlled trial focusing on patients who experienced post-extubation respiratory failure within the 7 days following extubation. Patients were classified in the NIV group or the high-flow nasal oxygen group according to oxygenation strategy used after the onset of post-extubation respiratory failure. Patients reintubated within the first hour after extubation and those promptly reintubated without prior treatment were excluded. The primary outcome was mortality at day 28 after the onset of post-extubation respiratory failure.

**Results:**

Among 651 extubated patients, 158 (25%) experienced respiratory failure and 146 were included in the analysis. Mortality at day 28 was 18% (15/84) using NIV alternating with high-flow nasal oxygen and 29% (18/62) with high flow nasal oxygen alone (difference, − 11% [95% CI, − 25 to 2]; *p* = 0.12). Among the 46 patients with hypercapnia at the onset of respiratory failure, mortality at day 28 was 3% (1/33) with NIV and 31% (4/13) with high-flow nasal oxygen alone (difference, − 28% [95% CI, − 54 to − 6]; *p* = 0.006). The proportion of patients reintubated 48 h after the onset of post-extubation respiratory failure was 44% (37/84) with NIV and 52% (32/62) with high-flow nasal oxygen alone (*p* = 0.21).

**Conclusions:**

In patients with post-extubation respiratory failure, NIV alternating with high-flow nasal oxygen might not increase the risk of death.

*Trial registration number*

The trial was registered at http://www.clinicaltrials.gov with the registration number NCT03121482 the 20th April 2017.

**Supplementary Information:**

The online version contains supplementary material available at 10.1186/s13054-021-03621-6.

## Background

In ICUs, around 20 to 30% of patients experience an episode of respiratory failure after extubation, although at the time of the decision to extubate they met all the usual criteria to be successfully separated from the ventilator [1, 2]. Nearly half of them eventually require reintubation with subsequently high mortality rates reaching 30–40% [1–5]. Consequently, an oxygenation strategy aimed at avoiding reintubation deserves consideration.

Prophylactic use of non-invasive ventilation (NIV) applied immediately after extubation may prevent post-extubation respiratory failure [1, 6–9]. By contrast, NIV used as rescue therapy to treat post-extubation respiratory failure could increase the risk of death by delaying reintubation [10]. The largest clinical trial conducted to date showed greater mortality with NIV than with standard oxygen even though reintubation rates were exactly the same [10]. The only difference explaining the deleterious effects of NIV was that the intubation delay was markedly longer than with standard oxygen. Thereby, the most recent international clinical practice guidelines suggest that NIV should not be used in the treatment of patients with established post-extubation respiratory failure [11]. However, NIV as rescue therapy may avoid reintubation in a number of cases, especially in hypercapnic patients with underlying chronic lung disease [7, 8, 12, 13], and recent large-scale clinical trials have shown that around 30 to 40% of patients with post-extubation respiratory failure are actually treated with NIV [1, 2]. High-flow nasal oxygen is increasingly used after extubation in order to prevent post-extubation respiratory failure [14–16], and could be used in case of post-extubation respiratory failure. Although its beneficial effects have been reported in treatment of acute respiratory failure [17], high-flow nasal oxygen has never been specifically studied for management of post-extubation respiratory failure, and the best oxygenation strategy in this setting remains unknown.

We recently conducted a randomized controlled trial showing that prophylactic use of NIV alternating with high-flow nasal oxygen immediately after extubation significantly decreased the risk of post-extubation respiratory failure as compared to high-flow nasal oxygen alone [1]. Based on this trial, we conducted a post-hoc analysis aimed at comparing the effects of NIV alternating with high-flow nasal oxygen vs. high-flow nasal oxygen alone on reintubation and mortality among patients experiencing post-extubation respiratory failure. We also aimed to compare reintubation rates and to identify risk factors associated with reintubation.

## Methods

### Study design and patients

Post-hoc analysis of a multicenter, randomized, controlled trial comparing prophylactic use of NIV alternating with high-flow nasal oxygen (i.e. NIV interspaced with high-flow nasal oxygen between NIV sessions) versus high-flow nasal oxygen alone immediately after extubation in 641 patients at high-risk of extubation failure in ICUs [1]. The present analysis focused on patients who experienced respiratory failure within the 7 days following extubation. Post-extubation respiratory failure was prospectively collected and was defined by the presence of at least two criteria among the following: respiratory rate > 25 breaths per minute, clinical signs suggesting respiratory distress, respiratory acidosis defined as pH < 7.35 units and PaCO_2_ > 45 mm Hg, and hypoxemia defined as FiO_2_ ≥ 50% to maintain SpO_2_ ≥ 92% or a PaO_2_/FiO_2_ ≤ 150 mm Hg. Hypoxemia was assessed using arterial blood gases performed at one hour, six hours, between 12 and 24 h and between 24 and 48 h following extubation, and then once a day until ICU discharge. For patients under standard oxygen, FiO_2_ was calculated according to the following formula: FiO_2_ = 0.21 + 0.03 per supplemental litre of oxygen [18].

Patients reintubated within the first hour after extubation and those who were promptly reintubated without prior specific treatment (i.e., who did not receive neither NIV nor high-flow nasal oxygen between the onset of respiratory failure and reintubation) were not retained in the analysis.

The original study was approved by the independent ethics committee of Poitiers (Ethics Committee Ouest III, Poitiers, France). Written informed consent was obtained from all patients or next of kin before inclusion.

### Treatment groups

The choice of oxygenation strategy to treat post-extubation respiratory failure was left to the physician's decision. Patients in whom NIV was continued or initiated after the onset of post-extubation respiratory failure were classified in the NIV group. Patients who were treated with high-flow nasal oxygen alone after onset of post-extubation respiratory failure were classified in the high-flow nasal oxygen group. If the decision was to use NIV, it was recommended to use a minimal pressure-support level of 5 cm H_2_O targeting a tidal volume around 6 to 8 ml/kg of predicted body weight, a positive end-expiratory pressure (PEEP) level between 5 and 10 cmH_2_O and a fraction of inspired oxygen (FiO_2_) adjusted to obtain adequate oxygenation (SpO_2_ ≥ 92%). If the decision was to use high-flow nasal oxygen, it was recommended to deliver a flow rate of 50 L/min and FiO_2_ adjusted to obtain adequate oxygenation (SpO_2_ ≥ 92%).

### Outcomes

The main outcome was mortality rates within the 28 days after the onset of post-extubation respiratory failure according to oxygenation strategy. Although reintubation within the 7 days following extubation was the primary outcome in the original study [1], we decided to choose mortality as primary outcome rather than reintubation given NIV could be associated with an increased risk of death without increased risk of reintubation compared with oxygen [10].

Secondary outcomes included reintubation rates within the first 48 h after the onset of respiratory failure and up until ICU discharge, the interval between the onset of respiratory failure and reintubation, length of stay in the ICU, mortality in the ICU and within 90 days following extubation. Severe respiratory failure leading to reintubation was defined by the presence of at least two criteria among the following: respiratory rate > 35 breaths per minute, clinical signs suggesting respiratory distress, respiratory acidosis defined as pH < 7.25 units and PaCO_2_ > 45 mm Hg, hypoxemia defined as FiO_2_ ≥ 80% to maintain SpO_2_ ≥ 92% or a PaO_2_/FiO_2_ ≤ 100 mm Hg.

### Statistical analysis

Continuous variables were expressed as mean ± standard deviation (SD) or median [interquartile range, IQR 25th–75th percentiles], and qualitative variables were expressed as number and percentage.

Mortality rates within the 28 days following post-extubation respiratory failure were compared between the NIV group and the high-flow nasal oxygen group by means of the χ^2^ test. Kaplan–Meier curves were plotted to assess the time from the onset of post-extubation respiratory failure to death and were compared by means of the log-rank test at day 28. As the effect of oxygenation strategy on mortality may be different according to PaCO_2_ level [7], a subgroup analysis was performed in patients with hypercapnia at the onset of respiratory failure (defined as PaCO_2_ > 45 mm Hg).

Secondary outcomes including reintubation rates were compared between the 2 groups by means of the χ^2^ tests or Fisher exact test for categorical variables and Student’s *t*-test or Wilcoxon test for continuous variables as appropriate. Kaplan–Meier curves were plotted to assess time from the onset of post-extubation respiratory failure to reintubation and were compared by means of the log-rank test at 48 h. A multiple logistic regression analysis was performed for reintubation in ICU with the use of a backward-selection procedure. All variables associated with reintubation with a *p* value of less than 0.20 after univariate analysis were entered into the maximal model. The results were presented as odds ratio (OR) with 95% confidence interval (95CI). A two-tailed p value of less than 0.05 was considered statistically significant. We used SAS software, version 9.4 (SAS Institute), for all the analyses.

## Result

Among the 651 patients extubated in the 30 participating ICUs, 158 (25%) experienced respiratory failure within the 7 days following extubation. After excluding 1 patient with missing data, 4 patients who were reintubated within the first hour after extubation, and 7 patients who were promptly reintubated without prior treatment by high-flow nasal oxygen or NIV, 146 patients with post-extubation respiratory failure were retained in the analysis including 84 patients treated with NIV alternating with high-flow nasal oxygen and 62 patients treated with high-flow nasal oxygen alone (Table 1). Interval between extubation and respiratory failure did not differ between the 2 groups (22 h in median in the NIV group [IQR 4–57] vs. 20 h in the high-flow oxygen group [IQR 5–47], *p* = 0.89). Among the 123 patients in whom blood gas measurement was obtained at the onset of post-extubation respiratory failure, 46 patients (37%) had hypercapnia. NIV was more frequently used in patients who had hypercapnia at time of post-extubation respiratory failure and in those who had already received NIV as preventive measure before the onset of respiratory failure.

**Table 1:** Comparison of patients treated with high-flow nasal oxygen alone and those treated with non-invasive ventilation (NIV) for post-extubation respiratory failure

	High-flow nasal oxygen (n = 62)	Non-invasive ventilation (n = 84)	*P* value
*Characteristics of the patients at admission*			
Age, years	71 ± 9	70 ± 9	0.59
Male sex, n (%)	40 (65%)	53 (63%)	0.86
Body-mass index, kg/m^2^	29 ± 7	28 ± 7	0.91
SAPS II at admission, points	60 ± 18	56 ± 17	0.22
Underlying chronic cardiac disease, n (%)	27 (44%)	38 (45%)	0.84
Underlying chronic lung disease, n (%)	23 (37%)	34 (40%)	0.68
Chronic obstructive pulmonary disease, n (%)	18 (29%)	25 (30%)	0.92
Acute respiratory failure as reason for intubation, n (%)	35 (56%)	54 (64%)	0.34
*Characteristics of the patients the day of extubation*			
SOFA score, points	4.5 ± 2.7	4.4 ± 2.8	0.84
Duration of mechanical ventilation, median (IQR), days	5 [3–11]	6 [4–10]	0.71
Weaning difficulty^a^, n (%)			0.20
-Simple weaning	36 (58%)	49 (58%)	
-Difficult or prolonged weaning	26 (42%)	35 (42%)	
Ineffective cough, n/n total (%)	15/58 (26%)	31/82 (38%)	0,14
Abundant secretions, n/n total (%)	30/57 (53%)	32/83 (39%)	0,10
Administration of steroids before extubation, n (%)	4 (6%)	13 (15%)	0,09
Prophylactic NIV after extubation, n (%)	7 (11%)	56 (67%)	** < 0.001**
*Characteristics at time of respiratory failure*			
Interval between extubation and respiratory failure, hours	20 [5–47]	22 [4–57]	0.89
Systolic arterial pressure, mm Hg	136 ± 21	135 ± 26	0.82
Diastolic arterial pressure, mm Hg	67 ± 13	66 ± 16	0.68
Heart rate, beats/min	90 ± 28	87 ± 35	0.58
Respiratory rate, breaths/min	27 [23–33]	30 [25–37]	0.07
Clinical signs suggesting respiratory distress, n (%)	20 (32%)	33 (39%)	0.38
SpO_2_, %	94 ± 7	95 ± 4	0.40
PaO_2_, mm Hg	83 ± 39	80 ± 24	0.66
PaO_2_/FiO_2_, mm Hg	181 ± 72	187 ± 76	0.70
pH, units	7.46 ± 0.06	7.41 ± 0.10	** < 0.01**
PaCO_2_, mm Hg	41 ± 9	48 ± 17	** < 0.01**
Hypercapnia (PaCO_2_ > 45 mm Hg), n/n total (%)	13/50 (26%)	33/73 (45%)	**0.03**
*Treatment duration between the onset of respiratory failure and recovery or reintubation, hours*			
Duration of high-flow nasal oxygen, hours	14 [2–54]	10 [1–33]	**0.04**
Duration of NIV, hours	0 [0–0]	12 [4–27]	** < 0.001**

*P* values indicated in bold were considered statistically significant (*P* < 0.05)

Continuous variables are given in mean ± standard deviation or median [interquartile range, IQR 25th–75th percentiles] according to their distribution

SAPS = Simplified Acute Physiology Score; SOFA = Sepsis-related Organ Failure Assessment; NIV = Non-Invasive Ventilation

^a^Weaning difficulty was defined as follows: simple weaning included patients extubated after success of the initial spontaneous breathing trial, difficult weaning included patients who failed the initial spontaneous breathing trial and were extubated within the 7 following days, and prolonged weaning included patients extubated more than 7 days after the initial spontaneous breathing trial

Ventilator settings used for the treatment of post-extubation respiratory failure using NIV were the following: a pressure-support level of 8 ± 3 cm H_2_O, a PEEP level of 5 ± 1 cm H_2_O, and FiO_2_ of 0.45 ± 0.16, resulting in a tidal volume of 8 ± 2 ml per kilogram of predicted body weight. Patients treated with high-flow nasal oxygen alone received a gas flow rate of 50 ± 5 L/min with FiO_2_ of approximately 0.49 ± 0.16.

After the onset of post-extubation respiratory failure and until reintubation or recovery, NIV was delivered for a median of 12 h [IQR 4–27] and high-flow nasal oxygen for 10 h ([IQR 1–33] between NIV sessions in the NIV group. High-flow nasal oxygen was delivered for 14 h [IQR 2–54] in the high-flow nasal oxygen group.

### Primary outcome

**Fig. 1:**
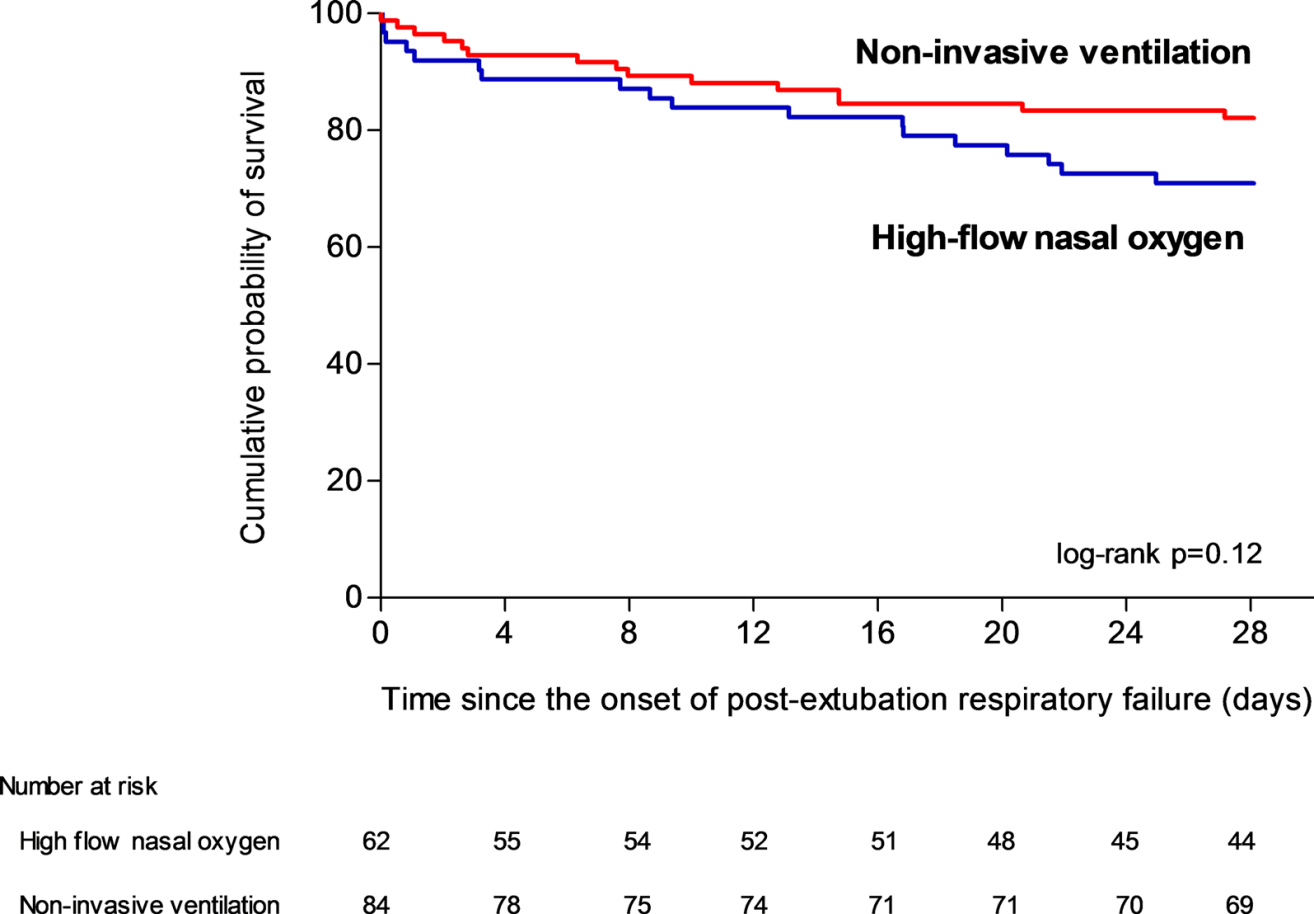
Kaplan–Meier analysis of time from the onset of post-extubation respiratory failure to death according to oxygenation strategy. Mortality rates at day 28 did not significantly differ between patients treated with high-flow nasal oxygen alone (blue line) and those treated with non-invasive ventilation (red line). Mortality at day 28 was 18% (15 out of 84 patients) with NIV and 29% (18 out of 62 patients) with high-flow nasal oxygen (difference, − 11% [95% CI, − 25 to 2]; *p* = 0.12 using log-rank test)

Mortality at day 28 was 18% (15 out of 84 patients) with NIV and 29% (18 out of 62 patients) with high-flow nasal oxygen alone (difference, − 11% [95% CI, − 25 to 2]; *p* = 0.11 using χ^2^ test and *p* = 0.12 using log-rank test) (Table 2 and Fig. 1).

**Table 2:** Comparison of outcomes between patients treated with high-flow nasal oxygen alone and those treated with non-invasive ventilation for post-extubation respiratory failure

	High-flow nasal oxygen (n = 62)	Non-invasive ventilation (n = 84)	Absolute difference, % (95% CI)	*P* value
*Primary outcome*				
Mortality at day 28, n (%)	18 (29%)	15 (18%)	− 11 (− 25 to 2)	0.11
*Secondary outcomes*				
Reintubation at 48 h, n (%)	32 (52%)	37 (44%)	− 7 (− 23 to 9)	0.37
Reintubation up until ICU discharge, n (%)	35 (56%)	40 (48%)	− 9 (− 24 to 7)	0.29
Mortality of reintubated patients, n (%)	11/35 (31%)	12/40 (30%)	− 1 (− 22 to 19)	0.89
Interval between the onset of respiratory failure and reintubation, hours	3.0 [1.3–10.5]	5.1 [1.8–18.0]	–	0.17
Length of stay in ICU, days	8 [4–14]	8 [5–17]	–	0.48
Mortality in ICU, n (%)	14 (23%)	16 (19%)	− 4 (− 17 to 9)	0.60
Mortality at day 90, n (%)	24 (39%)	26 (31%)	− 8 (− 23 to 8)	0.33

Continuous variables are given in mean ± standard deviation or median [interquartile range, IQR 25th–75th percentiles] according to their distribution

**Fig. 2:**
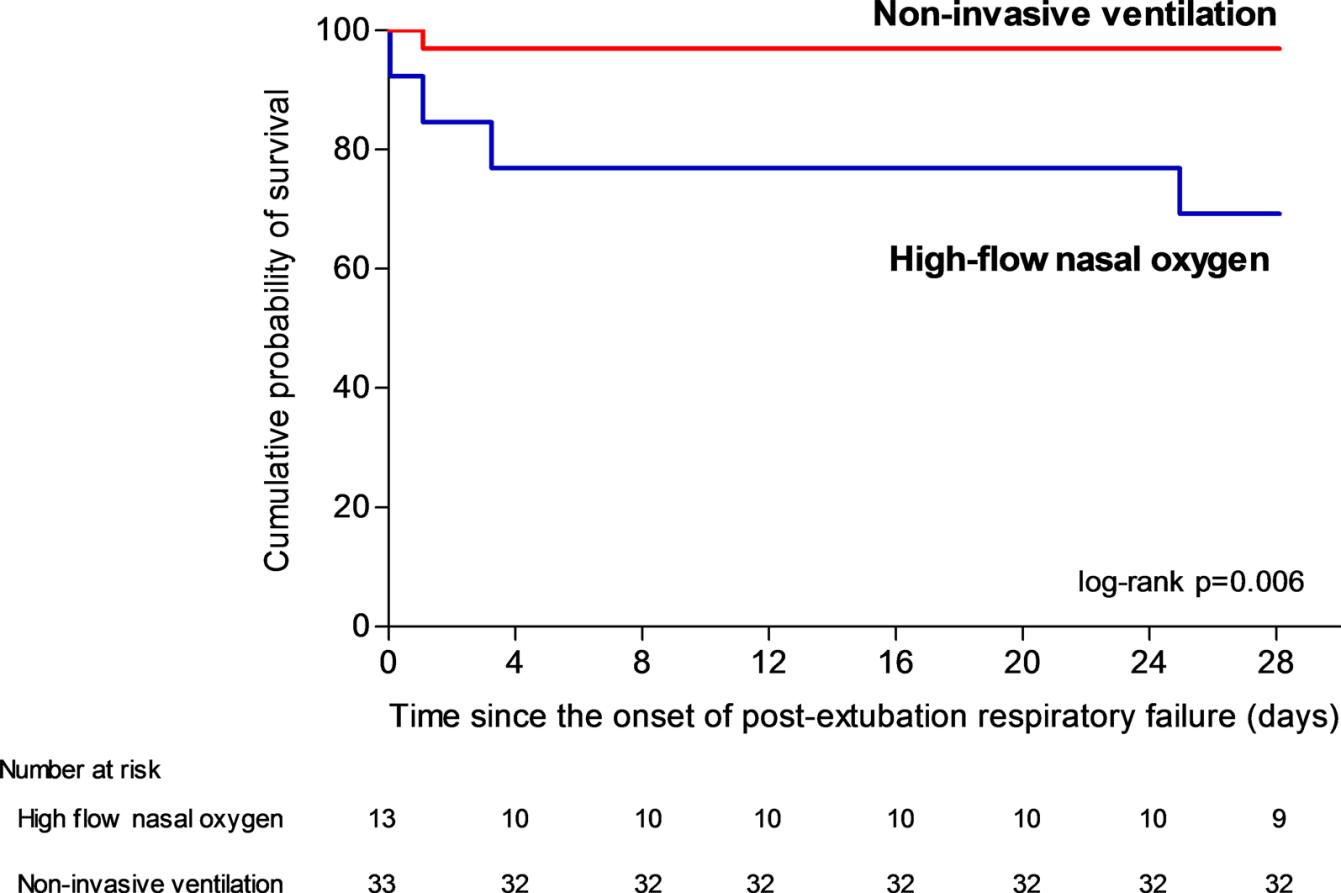
Kaplan–Meier analysis of time from the onset of post-extubation respiratory failure to death according to oxygenation strategy in the subgroup of patients with hypercapnia (PaCO_2_ > 45 mm Hg). Among the 46 patients with hypercapnia at the onset of respiratory failure mortality at day 28 was 3% (1 out of 33 patients) with NIV and 31% (4 out of 13 patients) with high-flow nasal oxygen alone (difference, − 28% [95% CI, − 54 to − 6]; *p* = 0.006 using log-rank test)

**Fig. 3:**
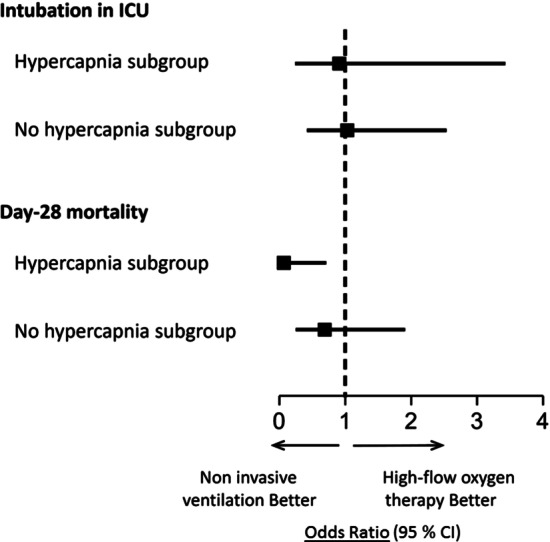
Odds Ratios for intubation in ICU and Day-28 Mortality (Primary Outcome) in subgroups of patients with hypercapnia (PaCO_2_ > 45 mm Hg) or no hypercapnia at time of post-extubation respiratory failure. Day-28 mortality was significantly lower with noninvasive ventilation than with high-flow nasal oxygen alone in the subgroup of patients with hypercapnia

Among the 46 patients with hypercapnia at the onset of respiratory failure, mortality at day 28 was 3% (1 out of 33 patients) with NIV and 31% (4 out of 13 patients) with high-flow nasal oxygen alone (difference, − 28% [95% CI, − 54 to − 6]; *p* = 0.02 using Fisher exact test and *p* = 0.006 using log-rank test) (Fig. 2). Among the 77 patients without hypercapnia, mortality at day 28 was 23% (9 out of 40 patients) with NIV and 30% (11 out of 37 patients) with high-flow nasal oxygen alone (difference, − 7% [95% CI, − 26 to 12]; *p* = 0.47 using χ^2^ test and *p* = 0.48 using log-rank test) (Fig. 3).

### Secondary outcomes

**Fig. 4:**
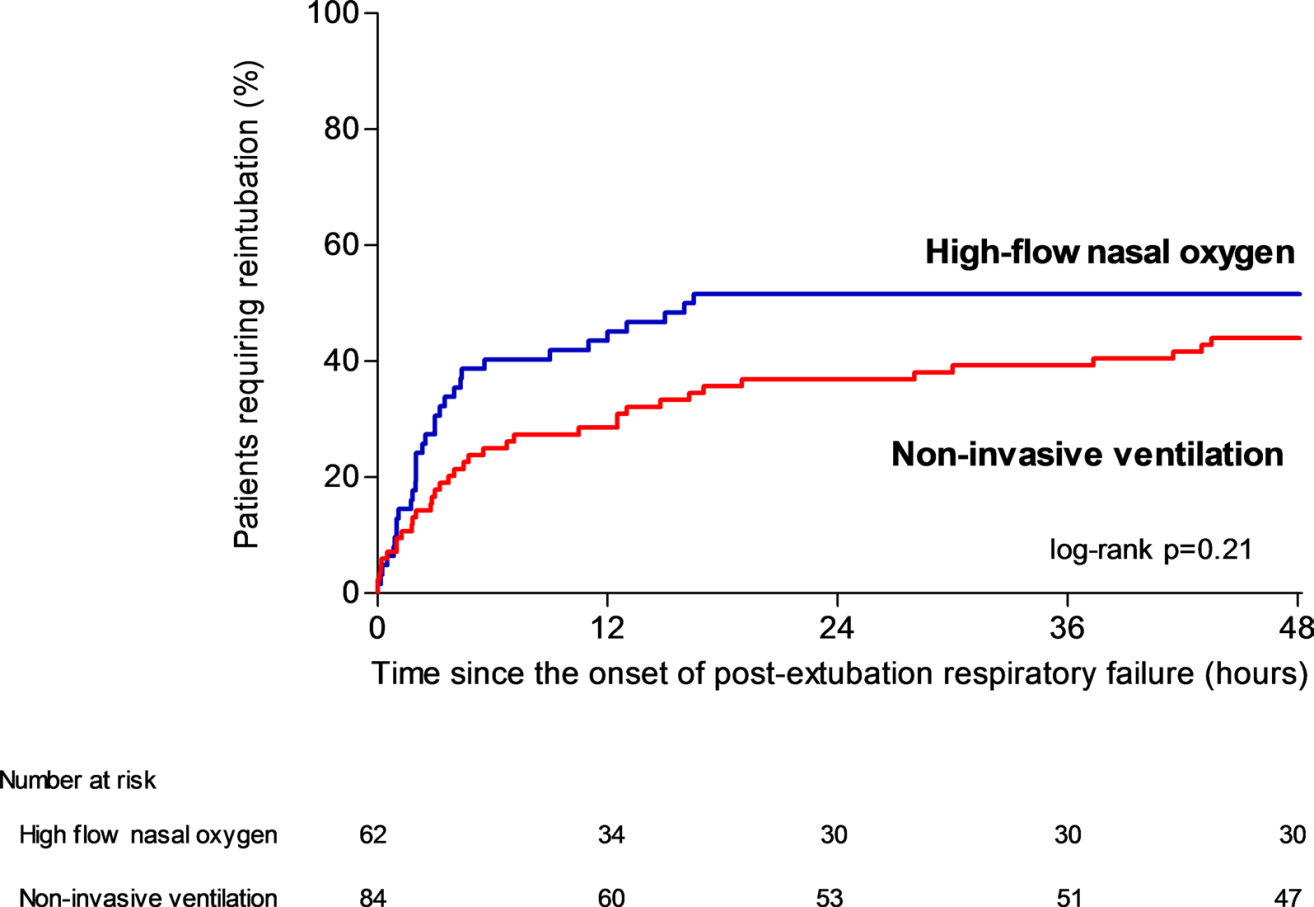
Kaplan–Meier analysis of time from the onset of post-extubation respiratory failure to reintubation according to oxygenation strategy. Reintubation rates did not significantly differ between patients treated with non-invasive ventilation (red bars) and those treated with high-flow nasal oxygen alone (blue bars). The reintubation rate within the first 48 h after the onset of post-extubation respiratory failure was 44% (37 out of 84 patients) with NIV and 52% (32 out of 62 patients) with high-flow nasal oxygen alone (difference, − 7% [95% CI, − 23% to 9%]; *p* = 0.21 using log-rank test)

The proportion of patients reintubated 48 h after the onset of post-extubation respiratory failure was 44% (37 out of 84 patients) with NIV and 52% (32 out of 62 patients) with high-flow nasal oxygen alone (difference, − 7% [95% CI, − 23% to 9%]; *p* = 0.37 using χ^2^ test and *p* = 0.21 using log-rank test) (Table 2 and Fig. 4). The interval between the onset of respiratory failure and reintubation did not significantly differ between groups: 5.1 h in median [IQR 1.8–18.0] in the NIV group and 3 h [IQR 1.3–10.5] in the high-flow nasal oxygen group (*p* = 0.17) (Table 2).

The reintubation rate in ICU was significantly lower in patients with hypercapnia than in those without hypercapnia: 37% (17 out of 46 patients) versus 57% (44 out of 77 patients), *p* = 0.03. Among the 46 patients with hypercapnia at the onset of respiratory failure, reintubation rates were 38% (5 out of 13 patients) with high-flow nasal oxygen alone versus 36% (12 out of 33 patients) with NIV (*p* = 0.89). Patients who required reintubation in ICU were younger, had lower body-mass index and were more likely to have abundant secretions than those who did not require reintubation. At time of respiratory failure, they had a higher heart rate, were less likely to have clinical signs suggesting respiratory distress and less frequently had hypercapnia (Table 3). After multivariable analysis, hypercapnia was the only variable significantly associated with not being reintubated. The probability of reintubation was 2.27 times higher (95% CI, 1.05 to 4.76; *p* = 0.04) in patients without hypercapnia as compared to patients with hypercapnia.

**Table 3:** Comparison between patients with post-extubation respiratory failure who required reintubation in ICU and those who were successfully treated without reintubation

	No reintubation (n = 71)	Reintubation (n = 75)	*P* value
*Characteristics of the patients at admission*			
Age, years	72 ± 9	69 ± 9	**0.049**
Male sex, n (%)	40 (56%)	53 (71%)	0.07
Body-mass index, kg/m^2^	30 ± 8	27 ± 6	**0.02**
Obesity, n (%)	30 (43%)	20 (27%)	**0.04**
SAPS II at admission, points	58 ± 18	57 ± 18	0.86
Underlying chronic cardiac disease, n (%)	33 (46%)	32 (43%)	0.64
Underlying chronic lung disease, n (%)	29 (41%)	28 (37%)	0.66
Acute respiratory failure as reason for intubation, n (%)	43 (61%)	46 (61%)	0.92
*Characteristics of the patients the day of extubation*			
SOFA score, points	4.1 ± 2.8	4.8 ± 2.7	0.11
Duration of mechanical ventilation, median (IQR), days	6 [3–13]	6 [3–13]	0.85
Weaning difficulty, n (%)			0.20
Simple weaning	36 (51%)	49 (65%)	
Difficult or prolonged weaning	35 (49%)	26 (35%)	
Ineffective cough, n/n total (%)	25/67 (37%)	21/73 (29%)	0.28
Abundant secretions, n/n total (%)	24/68 (35%)	38/72 (53%)	**0.04**
Administration of steroids before extubation, n (%)	12 (17%)	5 (7%)	0.05
Prophylactic NIV after extubation, n (%)	32 (45%)	31 (41%)	0.65
*Characteristics at time of ARF*			
Interval between extubation and respiratory failure, hours	20 [5–47]	22 [4–57]	0.89
Systolic arterial pressure, mm Hg	135 ± 25	136 ± 24	0.67
Diastolic arterial pressure, mm Hg	66 ± 14	67 ± 15	0.94
Heart rate, beats/min	82 ± 33	95 ± 30	**0.01**
Respiratory rate, breaths/min	39 ± 25	35 ± 22	0.38
Clinical signs suggesting respiratory distress, n (%)	38 (54%)	15 (20%)	** < 0.001**
SpO_2_, %	95 ± 4	94 ± 7	0.47
PaO_2_, mm Hg	83 ± 26	80 ± 36	0.59
PaO_2_/FiO_2_, mm Hg	196 ± 76	175 ± 72	0.18
pH, units	7,42 ± 0,08	7,44 ± 0,09	0.38
PaCO_2_, mm Hg	47 ± 13	43 ± 15	0.09
Hypercapnia (PaCO_2_ > 45 mm Hg), n/n total (%)	29/62 (47%)	17/61 (28%)	**0.03**
*Treatment of respiratory failure*			
Use of curative non-invasive ventilation, n (%)	44 (61%)	40 (53%)	0.29
Duration of administered treatment, hours	57 [28–86]	5 [2–16]	** < 0.001**
Duration of curative high-flow nasal oxygen hours	38 [16–72]	3 [1–10]	** < 0.001**
Duration of curative NIV, hours	10 [0–27]	2 [0–7]	**0.002**

*P* values indicated in bold were considered statistically significant (*P* < 0.05)

Continuous variables are given in mean ± standard deviation or median [interquartile range, IQR 25th–75th percentiles] according to their distribution

NIV = Non-invasive ventilation; SAPS = Simplified Acute Physiology Score

Values are given as mean ± standard deviation or median [interquartile range, 25th–75th percentiles]

Weaning difficulty was defined as following: simple weaning included patients extubated after success of the initial spontaneous breathing trial, difficult weaning included patients who failed the initial spontaneous breathing trial and were extubated within the 7 following days, and prolonged weaning included patients extubated more than 7 days after the initial spontaneous breathing trial

Patients who died in the ICU were less likely to exhibit clinical signs suggesting respiratory distress and had lower diastolic arterial blood pressure than those who were discharged alive from the ICU (Additional File 1).

## Discussion

In this post-hoc analysis of a randomized controlled trial focusing on patients who developed postextubation respiratory failure, mortality rates at day 28 did not differ between patients treated with NIV alternating with high-flow nasal oxygen and those treated with high-flow nasal oxygen alone. In the subgroup of patients with hypercapnia, mortality rate was significantly lower with NIV than with high-flow nasal oxygen alone. Patients with hypercapnia had a lower risk of reintubation than the others, regardless of the oxygenation strategy.

### Use of NIV to treat post-extubation respiratory failure

Few studies have assessed NIV in treatment of post-extubation respiratory failure. The first clinical trial was published in 2002 and included 81 patients with post-extubation respiratory failure randomly assigned to receive NIV or standard oxygen [19]. Reintubation rates were similar in the 2 groups (around 70%) and mortality rates in the ICU did not significantly differ (15% with NIV vs. 24% with standard oxygen, *p* = 0.34). In 2004, contrary to all expectations, another clinical trial including 221 patients with post-extubation respiratory failure showed that patients treated by NIV may have an increased risk of death as compared with standard oxygen [10]. Whereas reintubation rates were exactly the same between the 2 groups (48%), mortality rate in ICU was higher in the NIV group than in the standard oxygen group (25% vs. 14%, *p* = 0.048). In this study, patients treated with NIV were reintubated much later than those treated with standard oxygen (12 h in median vs. 2 h after the onset of respiratory failure), suggesting that NIV may worsen outcome by delaying reintubation. A meta-analysis performed in 2014 on these two studies indicated no benefit of NIV compared with standard oxygen [20]. No further large-scale clinical trial has been performed after these 2 studies and thereby, the most recent international clinical practice guidelines suggest that NIV should not be used in the treatment of patients with established post-extubation respiratory failure [11]. However, the experts have pointed out that both studies included few patients with chronic obstructive pulmonary disease (around 10%), so this recommendation may not apply to patients with underlying chronic lung disease who experience post-extubation respiratory failure, and that further studies are needed.

Unlike the clinical trial above-mentioned [10], we did not observe an increased risk of death in patients treated with NIV as compared with high-flow nasal oxygen. By contrast, we found that hypercapnic patients treated with NIV had even lower mortality than those treated with high-flow nasal oxygen alone. However, nearly 40% of the patients included in our study had underlying chronic lung disease compared to only 12% in the previous trial [10], and NIV may be particularly effective in this population. Moreover, the interval between NIV initiation and reintubation was markedly shorter in our study (5 h in median) than in theirs (12 h), and may explain that NIV was not associated with an increased risk of death. In our study, predefined criteria for intubation were precisely defined in order to minimize the risk of delayed intubation, and this may have helped to avoid potential deleterious effects of NIV in this setting. Furthermore, unlike previous studies [10, 19], all participating centers to our trial had extensive experience in NIV. Lastly, we used NIV alternately with high flow nasal oxygen and not NIV alone, which may have prolonged the beneficial effects of NIV during NIV breaks. However, all patients received high-flow nasal oxygen and NIV was the only additional treatment in the interventional group.

We found that patients with hypercapnia had a lower risk of reintubation than the others, regardless of the oxygenation strategy used. After multivariable analysis, hypercapnia at the onset of respiratory failure was the only factor independently associated with non-reintubation. These findings are in keeping with literature showing that patients with acute hypercapnic respiratory failure have lower intubation rates than non-hypercapnic patients [11, 21]. However, reintubation rates did not differ between patients treated with NIV and those treated with high-flow nasal oxygen alone, even in case of hypercapnia. Several physiological studies have shown that high-flow nasal oxygen decreased post-extubation respiratory drive and may help to reduce work of breathing and PCO_2_ almost as effectively as NIV [22–24]. Consequently, these two oxygenation strategies could have similar efficacy to reverse hypercapnia and to avoid reintubation in this setting. Nevertheless, hypercapnic patients treated with NIV had lower mortality than those treated with high-flow nasal oxygen alone. It could be hypothesized that NIV may promote alveolar recruitment and re-opening of atelectasis [25], improve clearance of tracheal secretions in case of weak cough [26], increase cardiac output and help treatment of cardiogenic pulmonary edema [27], and prevent apneas by delivering positive pressure [28]. Although this is an exploratory outcome focusing on a small-scale sample of patients, NIV use did not seem to have deleterious effects in this setting.

## Limitations

The main limitation of this study is the post-hoc nature of the analysis. However, reintubation and mortality rates observed in our study (51% and 21%, respectively) are almost exactly the same as those reported in the last clinical trial (48% and 21%, respectively) [10]. Reintubation rates were similar even though our patients were treated with high-flow nasal oxygen and not with standard oxygen as in previous trials [10, 19]. However, we only included patients at high-risk of extubation failure, which may explain why reintubation rates were not lower, despite a potentially more effective oxygenation strategy. Concerning the reference treatment, a recent guideline panel made a conditional recommendation for the use of high-flow nasal oxygen following extubation (moderate certainty) [29].

A major limitation is that the choice of oxygenation strategy was left to the physician's discretion. Characteristics of the patients were similar between the NIV group and the high-flow nasal oxygen group aside from the fact that NIV was more frequently used in case of hypercapnia, situation in which NIV is associated with high success rates [13], and in patients who had already received NIV as a preventive measure before the onset of respiratory failure. This could be explained by the fact that it is easier to continue an oxygenation strategy already in place than to initiate a new one, even though NIV is associated with particularly low success rates in this situation. Indeed, reintubation rates exceeding 70% have been when NIV is used as rescue therapy to treat patients who already received preventive NIV [7, 8]. Despite this potential imbalance between groups, we did not find worse outcomes with NIV than with high-flow oxygen alone and we even report a decreased risk of death in patients with hypercapnia.

## Clinical implications

Although the most recent international clinical practice guidelines suggest that NIV should not be used in the treatment of patients with post-extubation respiratory failure [11], our findings confirm that this oxygenation strategy is routinely used as rescue therapy to avoid reintubation in this setting. Indeed, recently published several large-scale randomized controlled trials have reported that around 30–40% of patients with post-extubation respiratory failure were treated with NIV [1, 2]. That could be explained by the fact that several studies have reported that NIV may help to avoid reintubation, especially in hypercapnic patients with underlying chronic lung disease [7, 8, 12]. These findings suggest that a large-scale randomized controlled trial should be conducted to better specify the most effective oxygenation strategy to treat established post-extubation respiratory failure in ICUs and potentially change clinical practice guidelines.

## Conclusion

In patients who experienced post-extubation respiratory failure, NIV alternating with high-high-flow nasal oxygen did not increase the risk of death in ICU as compared to high-flow nasal oxygen alone. Patients with hypercapnia at the onset of respiratory failure had lower reintubation rates than the others.

## Supplementary Information

**Additional file 1.** Comparison between patients who were discharged alive from ICU and those who died in ICUs after post-extubation respiratory failure.

## Data Availability

The datasets used and/or analysed during the current study are available from the corresponding author on reasonable request.
