# Supplementary material for: Non-invasive ventilation versus high-flow nasal oxygen for postextubation respiratory failure in ICU: a post-hoc analysis of a randomized clinical trial
Source: Crit Care. 2021 Jun 28;25:221. doi: 10.1186/s13054-021-03621-6 (PMC8236736; doi:10.1186/s13054-021-03621-6)
Supplement: Supplementary file 1 — Additional file 1. Comparison between patients who were discharged alive from ICU and those who died in ICUs after post-extubation respiratory failure. [file 13054_2021_3621_MOESM1_ESM.pdf]

Additional Table (Additional file 2): Comparison between patients who were discharged alive from ICU and those who died in ICUs after post-extubation respiratory failure.

	Discharged alive from ICU (n=116)	Died in the ICU (n=30)	P value
**Characteristics of the patients at admission**			
Age, years	70 ± 9	73 ± 9	0.1298
Male sex, n (%)	76 (65%)	17 (57%)	0.3689
Body-mass index, kg/m^2	29 ± 7	27 ± 7	0.1239
Obesity, n (%)	43 (38%)	7 (25%)	0.2067
SAPS II at admission, points	57 ± 18	60 ± 17	0.4278
Underlying chronic cardiac disease, n (%)	50 (43)	15 (50)	0.4981
Underlying chronic lung disease, n (%)	47 (41)	10 (33)	0.4722
Acute respiratory failure as reason for intubation, No. (%)	74 (64)	15 (50)	0.1675
**Characteristics of the patients the day of extubation**			
SOFA score, points	4,3 ± 2,8	5,0 ± 2,7	0.2191
Duration of mechanical ventilation, median (IQR), days	6 [3-11]	6 [4-9]	0.9421
Weaning difficulty, No. (%)			0.2035
Simple weaning	66 (57%)	19 (63%)	
Difficult or prolonged weaning	50 (43%)	11 (37%)	
Ineffective cough, n / n total (%)	34 (31%)	12 (40%)	0.3474
Abundant secretions, n / n total (%)	47 (43%)	15 (50%)	0.4772
Administration of steroids before extubation, n (%)	13 (11%)	4 (13%)	0.7462
Prophylactic non-invasive ventilation after extubation, n (%)	52 (45%)	11 (37%)	0.4211
**Characteristics at time of ARF**			
Interval between extubation and respiratory failure, hours	21 [4-55]	21 [5-47]	0.9710
Systolic arterial pressure, mm Hg	137 ± 24	130 ± 24	0.2207
Diastolic arterial pressure, mm Hg	68 ± 14	60 ± 15	**0.0063**
Heart rate, beats/min	87 ± 33	92 ± 29	0.4382
Respiratory rate, breaths/min	38 ± 24	34 ± 22	0.5124
Clinical signs suggesting respiratory distress, n (%)	48 (41%)	5 (17%)	**0.0121**
SpO_2, %	95 ± 5	93 ± 5	0.1086
PaO_2, mm Hg	82 ± 33	79 ± 20	0.7228
PaO_2/FiO_2, mm Hg	188 ± 76	171 ± 68	0.3921
pH, units	7,43 ± 0,09	7,41 ± 0,09	0.2626
$PaCO_2$, mm Hg	45 ± 14	46 ± 16	0.8860

Hypercapnia ($PaCO_2$ > 45 mm Hg), n / n total (%)	38/99 (38%)	8/24 (33%)	0.6464
**Treatment of ARF**			
Use of non-invasive ventilation as rescue therapy, n (%)	68 (59%)	16 (53%)	0.6015
Duration of NIV, hours	16 [2-46]	4 [1-10]	0.0614
Duration of high-flow nasal oxygen, hours	3 [0-18]	2 [0-9]	0.3376
Duration of treatment between the onset of respiratory failure and recovery or reintubation, hours	27 [5-67]	7 [2-19]	**0.0069**
Reintubation at any time in the ICU, n (%)	52 (45%)	23 (77%)	**0.0019**

Abbreviations: NIV = Non-invasive ventilation; SAPS = Simplified Acute Physiology Score.
Values are given as mean ± standard deviation or median [interquartile range, 25th- 75th percentiles]
Weaning difficulty was defined as following: simple weaning included patients extubated after success of the initial spontaneous breathing trial, difficult weaning included patients who failed the initial spontaneous breathing trial and were extubated within the 7 following days, and prolonged weaning included patients extubated more than 7 days after the initial spontaneous breathing trial.
